# Peptidylarginine deiminase 4 deficiency alleviates hypoxia/reoxygenation-induced cardiomyocyte injury

**DOI:** 10.1371/journal.pone.0330864

**Published:** 2025-09-10

**Authors:** Matthias Mand, Michelle Holthaus, Helmut R. Lieder, Petra Kleinbongard, Lenard Conradi, Thorsten Wahlers, Adnana Paunel-Görgülü

**Affiliations:** 1 Department of Cardiac Surgery, Faculty of Medicine and University Hospital Cologne, University of Cologne, Cologne, Germany; 2 Cardioprotection Unit, Institute for Pathophysiology, West German Heart and Vascular Center, University of Essen Medical School, University of Duisburg-Essen, Essen, Germany; INSERM, FRANCE

## Abstract

**Background:**

Cardiac ischemia reperfusion (I/R) injury is a serious consequence of reperfusion therapy for myocardial infarction (MI). Peptidylarginine deiminase 4 (PAD4) is a calcium-dependent enzyme that catalyzes the citrullination of proteins. In previous studies, PAD4 inhibition protected distinct organs from I/R injury by preventing the formation of neutrophil extracellular traps (NETs) and attenuating inflammatory responses. Here, we hypothesized that cardiomyocyte PAD4 expression may play a role in acute I/R injury.

**Methods:**

Infarct size was determined in isolated pressure constant-perfused hearts from WT and PAD4-deficient (PAD4^-/-^) mice. Additionally, extracellular reactive oxygen species (ROS) and cell viability were quantified in freshly isolated adult cardiomyocytes exposed to hypoxia followed by reoxygenation (H/R). Resistance to oxidative stress was proven in both genotypes by treatment of neonatal cardiomyocytes with hydrogen peroxide. Moreover, intracellular ROS formation, ATP production, mitochondrial membrane polarisation, caspase-3 activation, and cell viability were quantified after hypoxia followed by 4 h and 20 h of reoxygenation, respectively. The PAD4-specific inhibitor GSK484 was added before H/R or at reperfusion in certain experiments.

**Results:**

Infarct size was smaller in PAD4^-/-^ hearts following I/R when compared to the WT. Similarly, the viability of adult and neonatal PAD4^-/-^ cardiomyocytes was better preserved after H/R, accompanied by reduced ROS formation. PAD4 deficiency maintained mitochondrial integrity and protected neonatal cardiomyocytes against apoptosis. However, these cells did not exhibit resistance to hydrogen peroxide-induced cell death, indicating an unaltered antioxidative state. Whereas pharmacological PAD4 inhibition by GSK484 before H/R sustained intracellular ATP levels in WT cardiomyocytes, administration of GSK484 at reoxygenation did not. However, GSK484 significantly improved cardiomyocyte metabolic activity, regardless of the time of administration.

**Conclusions:**

Our study is the first to demonstrate that PAD4 expression in cardiomyocytes contributes to H/R injury independent of systemic immune responses and NETs. Consequently, PAD4 may serve as a therapeutic target to alleviate I/R injury.

## Introduction

Cardiovascular diseases (CVDs) are the leading cause of death worldwide [1]. According to the WHO, 32% of all global deaths were attributed to CVDs in 2019, whereas 85% of these deaths were due to myocardial infarction (MI) or stroke. The acute obstruction of an epicardial coronary artery due to plaque rupture of an atherosclerotic lesion leads to ST-segment elevation (STEMI) and permanent damage to the myocardium at risk if blood flow to the region of ischemia is not restored. The only way to salvage the myocardium at risk from infarction is timely reperfusion, which is nowadays realized through primary percutaneous coronary intervention (PCI). Paradoxically, while timely reperfusion demonstrably preserves cardiomyocyte viability, the reperfusion process itself is not without risk and may cause cardiomyocyte death contributing to up to 50% of the final infarct size [2,3]. Reperfusion-induced injury is a complex process accompanied by excessive production of reactive oxygen species (ROS), intracellular and mitochondrial calcium overload, both of which being well-established triggers of mitochondrial permeability transition pore (mPTP) opening [4]. With the mPTP opened, the cells are no longer able to maintain mitochondrial membrane potential resulting in rapid depletion of adenosine triphosphate (ATP) stores and cell death through oncosis and necrosis. Moreover, opening of the mPTP increases the permeability of the inner mitochondrial membrane promoting mitochondrial swelling, translocation of pro-apoptotic factors including cytochrome c into the cytosol and induction of apoptotic cell death [5,6]. The different clinical manifestations of reperfusion injury include irreversible lethal reperfusion injury, myocardial stunning, arrhythmias and microvascular obstruction [7]. Despite multiple pharmacological and interventional therapeutic strategies already tested in preclinical and clinical settings [3], there is still no effective treatment strategy to limit I/R. Therefore, the development of cardioprotective drugs that can be given before or at reperfusion is still a major unmet medical need [8].

Peptidylarginine deiminases (PADs) represent a family of five calcium-dependent isozymes (PAD1-PAD4 and PAD6) that mediate the posttranslational conversion of arginine into citrulline. PAD4 is the only member of the PAD family that carries a nuclear localization signal sequence [9]. It is primarily expressed in myeloid cells including neutrophils [10] and macrophages [11]. By the citrullination of nuclear histones, PAD4 promotes chromatin unfolding contributing to the formation of neutrophil extracellular traps (NETs) [12] and the regulation of gene expression [13]. PAD4 has been implicated in a substantial number of diseases including inflammation [14], cancer [15], rheumatoid arthritis [16], and I/R injury [17]. In this context, many studies have demonstrated that PAD4 inhibition can protect organs from ischemic injury in animal models, identifying NETs as the primary contributors to reperfusion injury [18–21]. Consequently, reduced infarct size has been reported after myocardial I/R injury in PAD4^-/-^ mice [19,21]. However, PAD4 expression has also been demonstrated in cardiac fibroblasts and cardiomyocytes [22], although little to no information is available regarding its function in non-myeloid cells.

In this study, we investigated the role of PAD4 in mouse hearts during I/R injury using an isolated perfused heart model, as well as in isolated cardiomyocytes, to exclude any involvement of immune cells.

## Materials and methods

### Mice

All animal procedures were approved by the local ethics committee (Bezirksregierung Köln; No.: 4.19.007; 4.22.024). Mice were housed at the University of Cologne. Global PAD4^-/-^ mice were generated as described previously [23]. PAD4^-/-^ mice lack exons 9–10 containing part of the PAD4 active site, as well as four additional residues that are essential for calcium binding, resulting in non-functional enzyme [24]. All mice were maintained in the local animal facility at a 12-h light/dark cycle and were given ad libidum access to standard diet and water.

The experimental protocols in isolated buffer-perfused mouse hearts and cardiomyocytes as well as the methods for the measurement of coronary flow (CF), left ventricular (LV) pressure, the quantification of infarct size, and cardiomyocyte viability were standard [25] and have been described in detail previously [26,27].

### Isolated heart preparation and protocols

Hearts from male mice (between 12 and 20 weeks of age) were isolated after cervical dislocation, quickly stopped in ice-cold saline and immediately mounted on a modified Langendorff-perfusion apparatus. Hearts were perfused at constant pressure (80 mmHg) with modified Krebs-Henseleit buffer, as described previously [27]. Heart rate was set to 500 beats/min by right atrial pacing. CF was measured with an inline ultrasonic flowprobe (TS410, Transsonic Systems Inc., Ithaca, NY, USA) above the aortic cannula. A water-filled custome-made cling-film balloon was inserted into the LV cavity and connected to a pressure transducer (Codan-PVB, Lensahn, Germany) to measure LV pressure. End-diastolic LV pressure, representing the pressure within the LV following the completion of diastolic filling, was set to 1–7 mm Hg at baseline by graded balloon inflation during the initial 5 min perfusion. Left ventricular diastolic pressure (LVDP), reflecting ventricular compliance, was calculated as the difference between peak and end-diastolic LV pressure. CF, end-diastolic, and peak LV pressure were continuously recorded (LabChart 8, AD Instruments Pty LTD, New South Wales, Australia). Hearts were allowed to stabilize for 10–20 min. CF and LVDP were calculated as mean values during the last min each of the stabilization period (baseline), at 5 and 25 min ischemia and at 10, 30 and 60 min reperfusion, respectively. Preparations with CF < 1.0 mL/min or >5.0 min or LVDP <60 mmHg at baseline were excluded. Three isolated heart preparations in total did not meet baseline criteria and were thus excluded from further analysis. After CF and LVDP were recorded at baseline, hearts were subjected to 30 min global zero-flow ischemia and 120 min reperfusion. The temperature of the perfusion buffer was monitored with probes in the aortic cannula throughout the experiment and kept between 37.5°C and 37.8°C by heat exchangers. Hearts were continuously warmed in a 37.5 to 37.7°C humidified chamber.

### Determination of infarct size

After termination of the protocol, hearts were frozen at −20°C and cut into transverse 1 mm thick slices. Infarcted tissue was demarcated by staining with 0.09 mol/L sodium phosphate buffer containing 1.0% triphenyl tetrazolium chloride (TTC) at 37°C for 5 min. Dehydrogenases in viable tissue react with the tetrazolium salt to form a red formazan compound. In contrast, dead tissue remains white showing the absence of living cells. Stained slices were photographed from both sides. The total slice area and the infarcted areas were quantified by computer-assisted planimetry (ImageJ 1.48v, National Institutes of Health, Bethesda, Maryland, USA), and infarct size was calculated as percent of the sum of left and right ventricular mass (% of ventricular mass), as described previously [28].

### Adult ventricular cardiomyocyte preparation

Male mice were sacrificed as described above, their hearts isolated and perfused with modified Tyrode buffer (in mM: 113.0 NaCl, 4.7 KCl, 0.6 KH_2_PO_4_, 0.6, Na_2_HPO4, 1.2 MgSO_4_, 12.0 NaHCO_3_, 10.0 KHCO_3_, 10.0 4-(2-hydroxyethyl)-1-piperazine ethanesulfonic acid, 30.0 taurine, 5.5 glucose, and 10.0 2,3-butanedione monoxime, pH 7.42 at 36.5 °C) at constant flow of 3 mL/min for 3 min. Liberase (Liberase TM Research Grade, Roche, Basel, Switzerland, 175 µg/ml), trypsin (75 µg/ml) and 12.5 µM CaCl_2_ were subsequently added to the perfusion buffer, and hearts were digested for 4 min. Atrial and connective tissue was removed and discarded, ventricles were sectioned, and cells were re-suspended in Tyrode buffer containing 10% bovine calf serum (Gibco, Thermo Fisher Scientific, Waltham, USA) and 12.5 µM CaCl_2_. Cardiomyocytes were isolated, separated from tissue residues by filtering through a nylon mesh filter (200 µm pore size, Millipore, Billerica, USA), and CaCl_2_ was slowly titrated at 20°C to a final concentration of 1 mM (5 steps of 10 min duration each). Cardiomyocytes were kept in modified Tyrode buffer (containing in mM: 125.0 NaCl, 5.4 KCl, 1.2 NaH_2_PO_4_, 20.0 4-(2-hydroxyethyl)-1-piperazine ethanesulfonic acid, 5.0 taurine, 15.0 glucose, 2.5 creatine, 0.5 MgCl_2_, and 1.0 CaCl_2_, gassed with 100% oxygen, pH 7.4) at normoxic conditions for 5 min before viability was determined at baseline.

### Simulation of H/R in adult ventricular cardiomyocytes

Freshly isolated adult ventricular cardiomyocytes from one single heart (WT or PAD4^-/-^) were divided into three aliquots, respectively. One aliquot served as a time control (TC), where cells where kept under normoxic conditions for 60 min. Two aliquots were subjected to a H/R protocol: After hypoxia was induced for 50 min by exposing cardiomyocytes to glucose-free, non-gassed buffer (in mM: 119.0 NaCl, 120.0 KCl, 5.0 4-(2-hydroxyethyl)-1-piperazine ethanesulfonic acid, 0.5 MgCl_2_, 0.9 CaCl_2_, 20.0 sodium lactate, pH 6.5) and sealing with mineral oil, cells were kept in solution where they sedimented. Reoxygenation was induced by removal of oil and glucose-free buffer and by adding reoxygenation buffer with an osmolality of 250 mosm/L (in mM: 88.0 NaCl, 5.4 KCl, 1.2 NaH_2_PO_4_, 12.0 NaHCO_3_, 20.0 4-(2-hydroxyethyl)-1-piperazine ethanesulfonic acid, 5.0 taurine, 15.0 glucose, 2.5 creatine, 0.5 MgCl_2_, and 1.0 CaCl_2_, gassed with 100% oxygen, pH 7.4) for 5 min. The cardiomyocyte viability of each aliquot was determined after H/R. The third aliquot was used to quantify extracellular ROS at baseline as well as after H/R.

### Isolation and culture conditions of neonatal cardiomyocytes

Cardiomyocytes were isolated from neonatal male and female mice (days 1–3) using the Pierce primary cardiomyocyte isolation kit (Thermo Fisher) according to manufacturer’s instructions. Briefly, freshly dissected hearts from decapitated mice were minced, incubated with Enzyme 1 and 2 for 35 min and washed twice with HBSS. The tissue was disrupted in Complete Dulbecco’s modified Eagle’s medium (DMEM) for Primary Cell Isolation (88287, Thermo Fisher) by pipetting up and down 25–30 times with a pipette fitted with a 1000 µl tip to generate a single cell suspension. Cardiomyocyte viability was ≥ 80% as assessed by trypan blue staining. Cells were cultured in Complete DMEM containing Cardiomyocyte Growth Supplement at a density of approximately 2.5 × 10^5^ cells/ cm^2^. Cells were used for experiments after five days in culture. The absence of immune cells with the potential to form NETs was confirmed by quantifying *Mpo* expression using real-time PCR. No *Mpo* expression was detected in cardiomyocyte cultures (not shown).

### Simulation of H/R in neonatal cardiomyocytes

To simulate H/R, cardiomyocytes were cultured in gluocose-free DMEM (11966025, Thermo Fisher) without FCS in a hypoxia chamber (Stemcell Technologies) with a gas mixture of 94% N_2_, 5% CO_2_ and 1% O_2_ at 37°C. Four hours later, the medium was replaced with DMEM for Primary Cell Isolation (88287, Thermo Fisher) supplemented with 10% FCS and 1% penicillin/ streptomycin and the cells were incubated under standard culture conditions (74% N_2_, 5% CO_2_, 21% O_2_) for up to 20 h at 37°C. Control cardiomyocytes were cultured in DMEM for Primary Cell Isolation under standard culture conditions in an incubator at 37°C.

When indicated, cardiomyocytes were exposed to hypoxia in the presence of 10 µM GSK484 (hydrochloride) (Cayman Chemical), a selective and reversible PAD4 inhibitor. Cardiomyocytes cultured in DMEM for Primary Cell Isolation supplemented with 10% FCS and 10 µM GSK484 under standard culture conditions at 37°C served as control.

### Determination of cardiomyocyte viability

Cell viability in neonatal cardiomyocytes was assessed using CellTiter 96 Aqueous One Solution Cell Proliferation assay (Promega) according to manufacturer’s instructions. Primary mouse cardiomyocytes (8 × 10^4^) were seeded in 96-well plates and cultured for five days. 20 h after H/R, 20 µl of the tetrazolium salt MTS (3-(4,5-dimethylthiazol-2-yl)-5-(3-carboxymethoxyphenyl)-2-(4-sulfophenyl)-2H-tetrazolium) was added to the culture medium (100 µl) and cells were further cultured for 4 h under standard culture conditions (74% N_2_, 5% CO_2_, 21% O_2_) at 37°C. In metabolically active cells MTS becomes presumably reduced by NADPH or NADH into a coloured formazan. To quantify formazan formation, which is directly proportional to the number of living cells, the absorbance was red at 490 nm using a multiplate reader (Victor X3, Perkin Elmer).

In parallel experiments as well as in adult ventricular cardiomyocytes, cell viability was additionally assessed by trypan blue staining using 0.5% trypan blue. For this, 300–700 adult cardiomyocytes per sample were analyzed in non-overlapping visual fields using light microscopy at 40 x magnification (Leica DMLB microscope, Leica, Bensheim, Germany). All samples were run in duplicate. Viability was expressed as the percentage of unstained cardiomyocytes over the total number of cells.

### Quantification of ROS

Dichlorodihydrofluorescein diacetate (DCFH-DA) was used to determine intracellular ROS after exposure of neonatal cardiomyocytes to H/R. For this, 10 µM DCFH-DA was added to the cells (8 × 10^4^/ 100 µl), followed by 45 min incubation in an incubator at 37°C. To determine the fluorescence intensity of 2 ^′^,7 ^′^-dichlorofluorescein (DCF), cells were washed twice with HBSS and the fluorescence intensity was determined using a multiplate reader (Victor X3, Perkin Elmer) at excitation and emission wavelengths of 485 and 535 nm, respectively. All samples were run in duplicate.

The Amplex Red Hydrogen Peroxide Assay (Invitrogen) was used to detect peroxides as a marker for ROS in the extracellular space at the end of normoxic incubation before hypoxia was induced and after hypoxia at the end of the 5 min reoxygenation period in freshly isolated adult ventricular cardiomyocytes. Amplex Red reacts in 1:1 stoichiometry with peroxides under catalysis by horseradish peroxidase (HRP) and produces highly fluorescent resorufin. Amplex UltraRed and 2 U/mol HRP were added to the normoxic, the hypoxic and the reoxygenation buffers, respectively. The supernatants of the normoxic buffer and of the reoxygenation buffer were collected and the yield of resorufin determined using a 96-well black plate and a F-7100 fluorescence spectrophotometer (Hitachi Hight-Tech, Tokyo, Japan) at 540 nm extinction and 580 nm emission wavelength, respectively. After collection of the supernatant the protein concentration of each remaining cardiomyocyte pellet was determined after cell lysis with 1 mM Tris/ 2% SDS lysis buffer using a commercial kit (Lowry method, Bio-Rad). ROS in the cardiomyocyte supernatant was then quantified in comparison with a H_2_O_2_ standard curve, normalized for protein concentration (100 μg) of the cardiomyocytes and expressed as extracellular ROS concentration.

### Induction of oxidative stress in neonatal cardiomyocytes

To induce oxidative stress, primary neonatal cardiomyocytes were treated with 200 µM or 400 µM hydrogen peroxide and cultured in DMEM for Primary Cell Isolation supplemented with 5% FCS and 1% penicillin/ streptomycin.

### Quantification of lactate dehydrogenase (LDH)

To quantify the leakage of LDH from damaged cells to the culture medium the LDH-GloTM Cytotoxicity Assay (Promega) was used by following the manufacturer’s instruction. Briefly, 10 µl supernatant were carefully collected and transferred to a microtube containing 990 µl LDH buffer. Then, 50 µl diluted supernatant was further transferred to a 96-well plate followed by the addition of 50 µl LDH detection reagent. Bioluminescence was recorded after 60 min incubation using Victor X3 multiplate reader (Perkin Elmer). All samples were run in duplicate. Cells treated with 10% Triton X-100 were used to generate maximum LDH release control and cytotoxicity was expressed as percentage cytotoxicity in relation to the maximum LDH release control.

### RNA isolation and gene expression analysis

Total RNA was isolated from neonatal cardiomyocytes or WT hearts using RNeasy Mini Kit (Qiagen) and cDNA synthesis was performed using High Capacity cDNA Reverse Transcription Kit (Applied Biosystems). For real-time PCR, the following gene-specific primer pairs were used: 5’-GGCTACACAACCTTCGGCAT-3’ and 5’-ATTCCCATCTCCCAGCTTCT-3’ for *Padi4*, 5’- GCCAAACTGAATCGCCAGA −3’ and 5’- ATGTTAAGAGCAGGCAAATCCA −3’ for *Mpo,* and 5’-CGACTTCAACAGCAACTCCCACTCTTCC-3’ and 5’-TGGGTGGTCCAGGG‐TTTCTTACTCCTT-3’ for *Gapdh*. Gene expression levels were determined using PowerUP SYBR Green PCR Master Mix (Applied Biosystems) according to the manufacturer’s recommended protocol with the following thermal cycling conditions: 2 min 50°C, 2 min 95°C, 40 cycles of 1 s 95°C and 30 s 60°C, and 4°C hold (QuantStudio 3 Real-Time PCR System, Applied Biosystems). All samples were run in triplicate. Expression of *Padi4* and *Mpo* was normalized to the GAPDH housekeeping gene.

### Detection of intracellular ATP

To estimate mitochondrial function, intracellular ATP levels were quantified in neonatal cardiomyocytes exposed to H/R using Luminescent ATP Detection Assay Kit (Abcam) according to the manufacturer’s protocol. In brief, cardiomyocytes (8 × 10^4^/ 100 µl) were mixed with 50 µl Detergent and shaked at 600 rpm for 5 min. After addition of 50 µl Substrate Solution, cells were further shaked at 600 rpm for 5 min followed by an incubation step for 10 min in the dark. All samples were run in duplicate. Bioluminescence was recorded using Victor X3 multiplate reader (Perkin Elmer).

### Measurement of mitochondrial membrane polarisation

Mitochondrial membrane potential (MMP) was evaluated using 5,5’,6,6’-tetrachloro-1,1’,3,3’-. tetraethylbenzi-midazolylcarbocyanine iodide (JC-1) staining. Neonatal cardiomyocytes were treated with JC-1 (2 µM) 4 h after reoxygenation, incubated for 30 min in the dark, followed by washing with PBS. In cells with intact MMP, JC-1 forms red to orange fluorescent (Ex = 585 nm, Em = 590 nm) aggregates in the mitochondrial matrix. With the loss of MMP, JC-1 is converted to the green fluorescent (Ex = 514 nm, Em = 529) monomeric form located to the cytoplasm. JC-1 fluorescence was quantified using flow cytometry (FACS Canto II, BD). A decrease in red to green fluorescence reflects the reduction of MMP after H/R.

### Western blotting

Samples containing 20 µg of protein were separated by 15% SDS-PAGE and transferred to a nitrocellulose membrane. After blocking of unspecific binding with 5% milk in TBST (TBS with 0.1% Tween-20), the membranes were incubated with polyclonal anti-Caspase-3 antibody (1:1000, 9662, Cell Signaling Technology) overnight at 4°C. Afterwards, the membranes were washed with TBST and incubated with HRP-conjugated goat anti-rabbit Immunoglobulins (Dako) for 1 h at room temperature. Protein bands were visualized using a chemiluminescent HRP substrate (Thermo Fisher) and imaged using the ChemiDoc Imaging System (Bio-Rad). After membrane stripping, membranes were reprobed with polyclonal rabbit anti-GAPDH antibody (1:1000, 2118, Cell Signaling Technology) in 5% BSA in TBST, followed by the incubation with HRP-conjugated goat anti-rabbit Immunoglobulins (Dako). Protein expression was normalized to that of the loading control GAPDH.

### Immunofluorescence

Neonatal cardiomyocytes were seeded on gelatin-coated coverslips and subjected to H/R as mentioned before. Then, cells were fixed with 4% paraformaldehyde, rinsed with PBS, and further incubated with blocking buffer consisting of 5% normal goat serum and 0.3% TritonX-100 in PBS for 1 h to block non-specific binding sites. After overnight incubation with rabbit anti-cleaved caspase-3 antibody (1:400, 9661, Cell Signaling Technology) or with rabbit anti-histone H3 (citrulline R2 + R8 + R17) antibody (1:500, ab5103, Abcam) diluted in blocking buffer at 4°C, samples were incubated for 1 h with Alexa Fluor 488-conjugated anti-rabbit IgG antibody (1:1000, 4412, Cell Signaling Technology). Cells were rinsed in PBS, counterstained with DAPI and mounted with Dako fluorescent mounting medium (Dako). Slides were imaged using an inverted microscope (Eclipse Ti-U 100, Nikon) and the number of cleaved caspase-3-positive cells was calculated using Image J software. To compare histone H3 citrullination between samples, all images were taken using the same exposure time.

For the detection of immune cells in WT hearts isolated at baseline, paraffin-embedded heart tissue sections were deparaffinized, rehydrated and antigen retrieval was performed using citrate buffer for 2 h. Sections were permeabilized using 0.5% Triton X-100 in TBS and blocked in TBS containing 5% goat serum and 0.1% TritonX-100 for 1 h. Sections were further stained with rabbit anti-histone H3 (citrulline R2 + R8 + R17) antibody (1:200, ab5103, Abcam) overnight at 4°C, followed by incubation with goat anti-rabbit Alexa Fluor 488-conjugated antibody (1:1000, 4412, Cell Signaling Technology) for 1 h at room temperature. Slides were counterstained with DAPI and embedded in fluorescence mounting medium (DAKO). Image acquisition was performed using an Eclipse Ti-U 100 microscope and NIS Element software package (Nikon).

### Quantification of neutrophils by flow cytometry

To assess neutrophil heart tissue infiltration under baseline conditions, WT hearts were minced and enzymatically digested as previously reported [29]. Dissociated cells were suspended in PBS supplemented with 2% FCS and 1 mM EDTA, pH 8.0 and stained with Violet 510 Viability Stain (Cell Signaling Technology) for 30 min on ice, followed by incubation with anti-mouse CD16/32 antibody (101319, Biolegend) to block Fc receptors. Then, the following fluorochrome-labeled antibodies were added: PerCP-Cy5.5-conjugated CD45 (1:80, 103131, Biolegend), APC-Cy7-conjugated CD11b (1:80, 101225, Biolegend), and FITC-conjugated Ly6G (1:200, 127605, Biolegend). Data were acquired on FACS Canto II (BD Biosciences) and data analysis was performed using FlowJo v10.9.1 (Tree Star).

### Statistical analysis

Data analysis was performed using GraphPad Prism (version 20.1.2, San Diego, CA, USA). Values were tested for a Gaussian distribution using the Shapiro-Wilk normality test. For comparisons of two groups unpaired two-tailed Student’s t test was used. Differences between more than two groups were compared using ordinary one-way ANOVA followed by Tukey’s multiple comparison test. Differences between groups of two independent variables were determined using two-way ANOVA and Tukey’s post hoc test or using a two-way ANOVA for repeated measures followed by Fischer’s LSD post hoc test. All data are presented as means ± SD. Differences were considered statistically significant at the level of *p* < 0.05.

## Results

### PAD4 deficiency reduces I/R- and H/R-induced damage in ventricular cardiomyocytes

**Fig 1:**
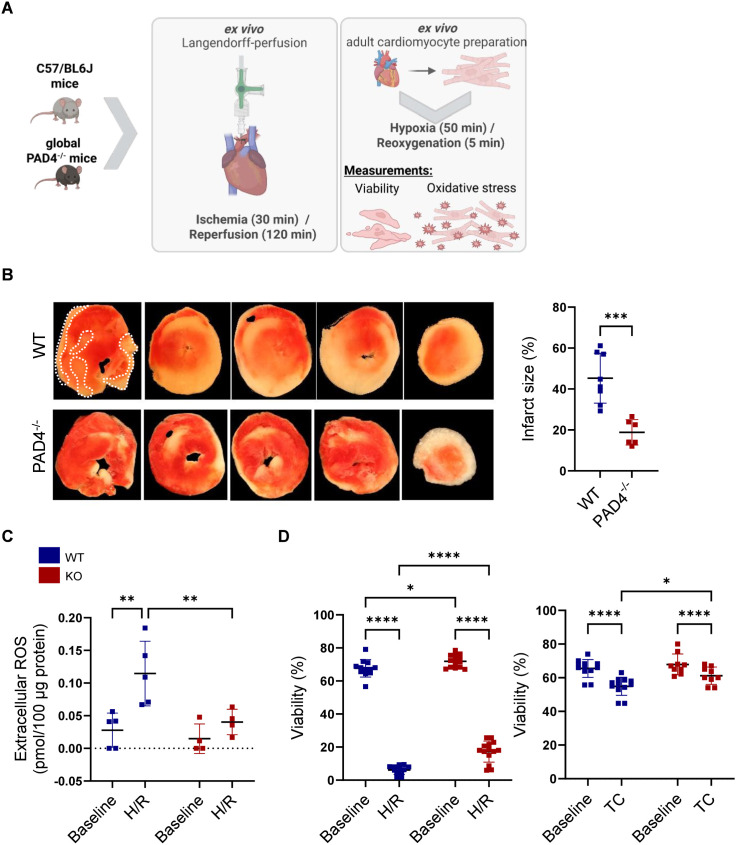
Impact of PAD4 on I/R- and H/R-induced cardiomyocyte damage. **(A)** Experimental design and protocols in whole hearts and freshly isolated adult cardiomyocytes (Created in BioRender. https://BioRender.com/txy6256). **(B)** Isolated pressure constant-perfused WT (n = 8) and PAD4^-/-^ (n = 6) hearts were exposed to 30 min ischemia followed by 120 min of reperfusion. Infarcted area, which appears pale, was quantified using TTC staining and is expressed as the percentage of the sum of left and right ventricular mass. Representative images are depicted. In one image, the infarct region is indicated by the dotted white line. **(C)** Quantification of extracellular ROS in freshly isolated adult WT (n = 5) and PAD4^-/-^ (n = 4) cardiomyocytes at baseline and after exposure to hypoxia for 50 min followed by 5 min of reoxygenation (H/R). **(D)** Cardiomyocyte viability at baseline and after H/R or time control (TC, without H/R) determined by trypan blue exclusion, respectively (n = 9-12 for WT; n = 12-14 for PAD4^-/-^). *p < 0.05; **p < 0.01; ***p < 0.001; ****p < 0.0001 (unpaired t test in B, two-way ANOVA for repeated measurements with Fischer’s LSD post hoc test in C and **D)**.

Previous studies have demonstrated that PAD4 activity contributes to I/R injury by exacerbating inflammation and NETs formation [18–20]. To investigate the role of PAD4 in cardiac cells excluding immune cells, global I/R was performed to isolated hearts from PAD4^-/-^ and WT mice. The experimental protocols for I/R and in vitro studies with adult cardiomyocytes are summarized in Fig 1A. As we previously reported, baseline heart function does not differ between the two genotypes [30]. In addition, baseline values for CF and LVDP were not different between groups (S1 Table). After 120 min of reperfusion, infarct size was quantified by TTC staining. We found infarct size in PAD4^-/-^ hearts to be significantly smaller than in WT hearts (Fig 1B). Accordingly, extracellular ROS, quantified in the supernatant of freshly isolated adult cardiomyocytes exposed to H/R, were highest in WT cardiomyocytes and only a slight, not significant increase in ROS production was observed in PAD4^-/-^ cardiomyocytes when compared to baseline ROS levels (Fig 1C). The yield of viable adult PAD4^-/-^ cardiomyocytes was higher at baseline but declined markedly in both genotypes following simulated H/R conditions. Nevertheless, the percentage of viable PAD4^-/-^ cardiomyocytes was significantly higher when compared to WT cells (Fig 1D, left panel). Incubation of cells under normoxic condition for the same time (TC) also reduced cell viability, albeit to a lesser extent, again resulting in a higher number of viable PAD4^-/-^ cardiomyocytes compared to WT (Fig 1D, right panel). As neutrophils capable of releasing NETs were demonstrated to be nearly undetectable under baseline conditions (S1 Fig), our findings clearly indicate that PAD4 mediates I/R- and H/R-induced cardiomyocyte injury in a NETs-independent manner.

### PAD4 does not influence the sensitivity towards oxidative stress

**Fig 2:**
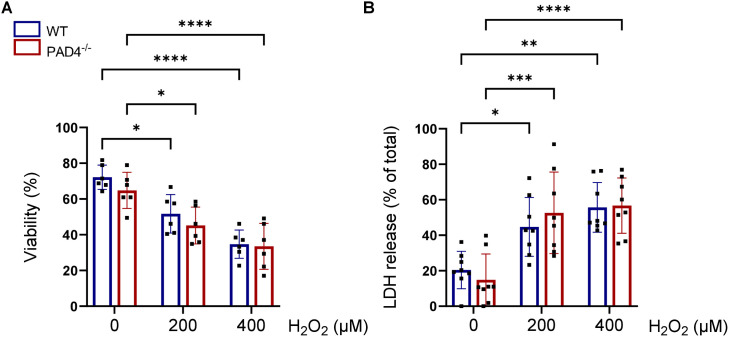
Impact of PAD4 on oxidative stress-related damage. Cultured cardiomyocytes isolated from neonatal WT (n = 6-8) and PAD4^-/-^ (n = 6-8) hearts were treated with 200 µM or 400 µM hydrogen peroxide. Cell viability (A, trypan blue) and LDH release (B) were quantified after 20 h. *p < 0.05; **p < 0.01; ***p < 0.001; ****p < 0.0001 (two-way ANOVA with Tukey’s post hoc test).

In order to investigate if PAD4 impacts the sensitivity against ROS, we subjected cultured neonatal WT and PAD4^-/-^ cardiomyocytes to 200 µM and 400 µM hydrogen peroxide. Compared to the control groups, viability significantly decreased in the presence of hydrogen peroxide (Fig 2A) ongoing with increased LDH release (Fig 2B). Notably, no differences were observed between WT and PAD4^-/-^ cardiomyocytes, suggesting that PAD4 deficiency does not reduce susceptibility to oxidative stress in neonatal mouse cardiomyocytes.

### PAD4 deficiency diminishes H/R-induced mitochondrial dysfunction and apoptosis in neonatal cardiomyocytes

**Fig 3:**
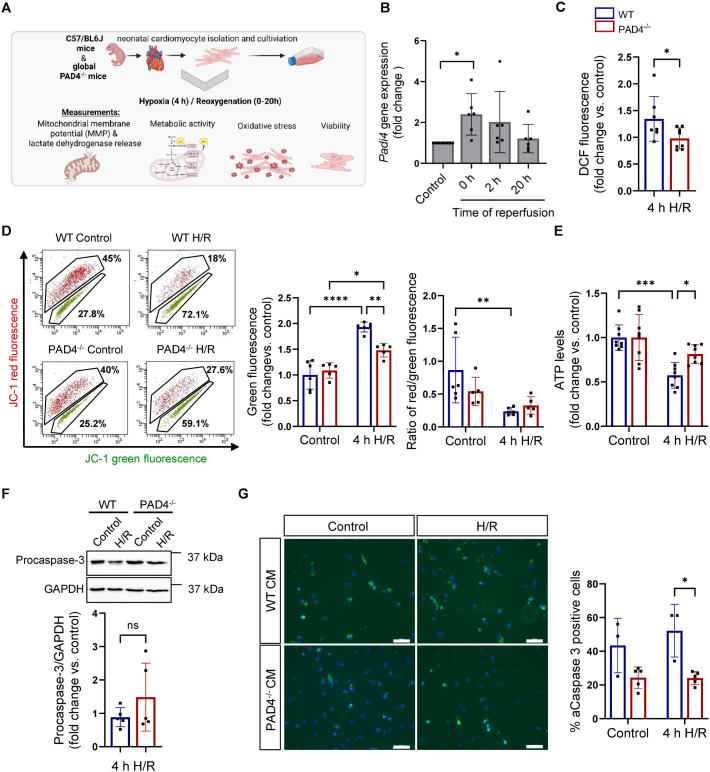
Effect of PAD4 deficiency on H/R-induced mitochondrial dysfunction. **(A)** Experimental workflow and analyses in cultured neonatal cardiomyocytes following H/R (Created in BioRender. https://BioRender.com/d8sqjzg). **(B)** *Padi4* expression in WT cardiomyocytes was analyzed by real-time PCR (n = 6). **(C)** Quantification of intracellular ROS in WT (n = 7) and PAD4^-/-^ (n = 8) cardiomyocytes after 4 h of reoxygenation. **(D)** Flow cytometry dot plots showing the gating of red fluorescent JC-1 aggregates and green fluorescent JC-1 monomers in cardiomyocytes after 4 h of reoxygenation. JC-1 monomers, indicating loss of mitochondrial membrane potential, as well as the ratio of red to green fluorescence after JC-1 staining were quantified (n = 6 for WT; n = 5 for PAD4^-/-^). **(E)** Quantification of intracellular ATP levels 4 h after reoxygenation (n = 8 per genotype). **(F)** Quantification of intracellular procaspase-3 levels in WT (n = 5) and PAD4^-/-^ (n = 5) cardiomyocytes exposed to hypoxia followed by 4 h of reoxygenation. Representative western blot with GAPDH as loading control. **(G)** Quantitative analysis of active caspase-3 (aCaspase-3) in cardiomyocytes after 4 h of reoxygenation. Representative immunofluorescence for aCaspase-3 (green) with DAPI-stained nuclei (blue) from WT (n = 3) and PAD4^-/-^ (n = 5) cardiomyocytes (CM) is shown. Scale bar: 50 µm. *p < 0.05; **p < 0.01; ***p < 0.001; ****p < 0.0001 (one-way ANOVA with Tukey’s post-hoc test in B; two-way ANOVA with Tukey’s post-hoc test in D, E, G; unpaired t test in C,F).

Based on these findings, we assumed that PAD4 may ameliorate I/R injury through mechanisms independent on antioxidant activity. To gain deeper insight into the role of PAD4 in cardiomyocytes, we first confirmed its activation in these cells by exposing neonatal cardiomyocytes to 4 h of hypoxia followed by up to 20 h of reoxygenation (H/R) (Fig 3A).

**Fig 4:**
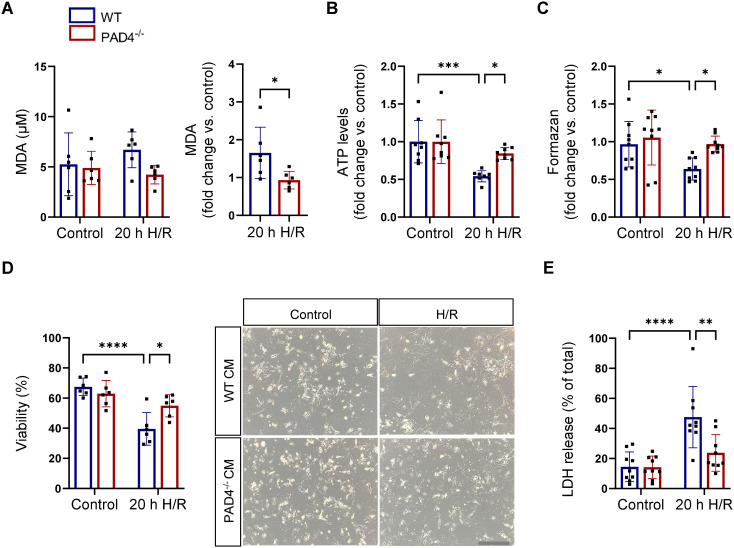
Effect of PAD4 on cardiomyocyte viability following H/R. **(A)** Quantification of MDA levels in WT (n = 6) and PAD4^-/-^ (n = 6) cardiomyocytes after 20 h of reoxygenation. **(B)** Quantification of intracellular ATP levels (n = 8 per genotype). **(C)** Quantification of formazan indicating metabolic activity (n = 10 per genotype). **(D)** Quantification of cell viability by trypan blue staining (n = 6 per genotype). Representative images of culture cardiomyocytes after 4 h of oxygen deprivation and 20 h of reoxygenation are shown. Scale bar: 250 µm. **(E)** Quantification of extracellular LDH levels (n = 9 per genotype). *p < 0.05; **p < 0.01; ***p < 0.001; ****p < 0.0001 (two-way ANOVA with Tukey’s post-hoc test in B-E; unpaired t test in **A)**.

We found significant upregulation of *Padi4* expression in WT cardiomyocytes immediately following hypoxia, which began to decline during reoxygenation (Fig 3B). Moreover, upregulation of PAD4 on transcriptional level was accompanied by a prominent increase in histone H3 citrullination, reflecting PAD4 activity (S2 Fig). *Padi4* ablation in cardiomyocytes was associated with diminished ROS formation after 4 h of reoxygenation (Fig 3C), indicating that PAD4 activity exerts an impact on intracellular ROS formation. As ROS strongly contribute to mPTP opening [4] we next measured mitochondrial membrane potential by JC-1 staining. According to reduced ROS amounts, PAD4^-/-^ cardiomyocytes displayed a significant decrease in green fluorescent JC-1 monomers compared to WT cells indicating more mitochondria with intact membrane potentials. Furthermore, the red to green ratio significantly decreased in WT cardiomyocytes, but not in PAD4^-/-^ cardiomyocytes, after 4 h of reoxygenation (Fig 3D). Consequently, strong ATP depletion was observed in WT cells after H/R, whereas ATP levels were largely sustained in PAD4^-/-^ cardiomyocytes (Fig 3E). Given that opening of the mPTP facilitates the release of pro-apoptotic factors to the cytosol and apoptosome formation, we further investigated the activation of caspase-3 to prove if PAD4 impacts apoptotic cell death. Western blot analysis revealed higher amounts of inactive, procaspase 3 in PAD4^-/-^ cardiomyocytes after H/R, though this increase was not statistically significant (Fig 3F). Detection of cleaved (= activated) caspase-3 by immunofluorescence and subsequent quantification demonstrated impaired caspase-3 activation in cells with compromised PAD4 activity (Fig 3G). These results are in line with increased mitochondrial integrity in these cells, and strongly affirm that PAD4 restricted to cardiomyocytes contributes to H/R injury. Indeed, after 20 h of reoxygenation, we found ameliorated lipid peroxidation, higher ATP levels ongoing with increased viability and reduced cell death in PAD4^-/-^ cardiomyocytes compared to WT cardiomyocytes (Fig 4A–4E).

### Pharmacological inhibition of PAD4 preserves cardiomyocyte viability

**Fig 5:**
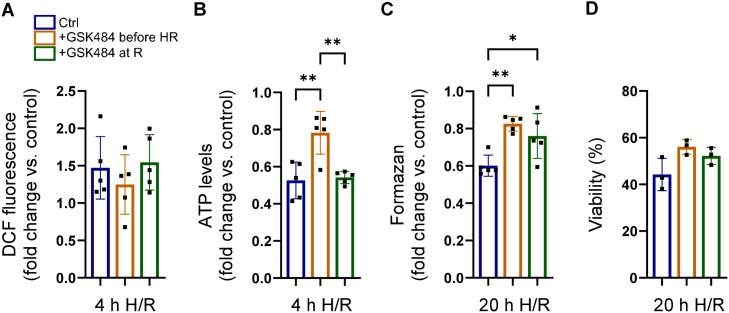
Impact of pharmacological PAD4 inhibition on cardiomyocyte survival following H/R. Cultured neonatal WT cardiomyocytes were treated with the selective PAD4 inhibitor GSK484 (10 µM) 2 h before hypoxia (+GSK484 before H/R) or at the onset of reperfusion (+GSK484 at R), respectively. WT cells with preserved PAD4 activity were used as control (Ctrl). Intracellular ROS (A, n = 5) and ATP levels (B, n = 5) were quantified after 4 h of reoxygenation. Formazan levels (C, n = 5) and the number of cells negative for trypan blue (D, n = 3) were determined after 20 h of reoxygenation. *p < 0.05; **p < 0.01 (one-way ANOVA with Tukey’s post hoc test).

Our results shown above suggest that pharmacological PAD4 inhibition may protect cardiomyocytes in the infarcted heart against reperfusion-induced injury. To determine if PAD4 inhibition might alleviate H/R-induced cell death, we treated neonatal WT cardiomyocytes with the reversible PAD4-selective inhibitor GSK484 two hours before the onset of hypoxia. GSK484 was demonstrated to inhibit PAD4 activity and histone H3 citrullination (S2 Fig). In parallel experiments, the inhibitor was administrated after hypoxia at the beginning of reoxygenation. There were no notable differences in ROS production between the different groups after 4 hours of reoxygenation (Fig 5A).

While pre-incubation of cells with GSK484 increased cellular ATP levels and metabolic activity (i.e., formazan formation) at 4 h and 20 h after reoxygenation, respectively (Fig 5B,C), it failed to augment cell viability determined by trypan blue exclusion (Fig 5D). Moreover, the protective effects were largely abolished when the inhibitor was applied at reperfusion, indicating that complete inhibition of PAD4 enzymatic activity is a prerequisite for preventing H/R-induced cardiomyocyte damage (Fig 5B–5C). Viewed together, these data suggest that timely administration of PAD4 inhibitors may exert an unanticipated cardioprotective effect by ameliorating mitochondrial dysfunction and improving viability of cardiomyocytes exposed to H/R.

## Discussion

Infarct size following MI remains a major determinant of patients’ outcome and is strongly associated with heart failure development and mortality. Among patients undergoing PCI after STEMI, every 5% increase in infarct size is linked to a 20% increase in mortality risk [31]. While timely reperfusion limits cardiomyocyte death, it causes additional cell death contributing to final infarct size. To date, no studies have specifically addressed the function of PAD4 in cardiomyocytes. To our knowledge, this is the first study to demonstrate that genetic ablation of PAD4 results in reduced I/R- and H/R-induced cardiomyocyte death, independent of NETs release. By performing in vitro experiments, we further demonstrate that loss of PAD4 in cardiomyocytes protects against mitochondrial dysfunction, thereby reducing apoptotic cell death following H/R.

PAD4 was firstly described to trigger the release of NETs from activated neutrophils as part of the innate immune system [32]. Although during the last decade an additional role of PAD4 in I/R injury has been suggested [19,20,33], very little is known about the underlying mechanisms. By subjecting mice with global PAD4 knockout to I/R, Savchenko *et al.* found reduced NETs formation in myocardial tissue, smaller infarct sizes, and improved heart function. Additionally, the authors suggested that lack of NETs during reperfusion results in cardioprotective effects [19]. However, our findings clearly demonstrate that protection against I/R-induced cardiac injury in *Padi4*-ablated mice is not solely attributable to NETs deficiency, as it persists even in the absence of neutrophils and NETs. Although cardiac baseline *Padi4* expression was reported to be very low compared to its expression levels found in neutrophils [34], the *Padi4* gene becomes rapidly upregulated during hypoxia in a HIF-1α-dependent manner [35]. This aligns with our observations showing *Padi4* upregulation following exposure of neonatal cardiomyocytes to hypoxia, accompanied by a concomitant and robust upregulation of PAD4 activity, as evidenced by increased abundance of citrullinated histone H3. The decrease in cellular ATP levels during hypoxia along with the resulting calcium elevation [36] enhance PAD4 activity manifold through calcium-induced conformational changes [37]. Surprisingly, PAD4 activity did not persist during the reperfusion phase. This finding may be explained by a ROS-dependent inhibition of PAD4 activity [38] as well as by the restoration of ATP and re-establishment of intracellular calcium levels during reperfusion [39].

Several sources of ROS within cardiomyocytes have been proposed including the mitochondrial electron transport chain, NADPH oxidase (NOX), and xanthine oxidase, whereby the electron transport chain is considered to represent the predominant source [40–42]. The high abundance of mitochondria in cardiomyocytes contributes to their enhanced sensitivity to oxidative stress damage [43]. Our results demonstrate that PAD4 deficiency in cardiomyocytes alleviates H/R-induced ROS generation, preserves mitochondrial integrity, and limits caspase-3 activation. These findings highlight PAD4’s role in apoptosis [44], although additional cell death pathways like necrosis, necroptosis, ferroptosis, and pyroptosis involved in myocardial I/R injury [45–48], might need further consideration. As PAD4^-/-^ cardiomyocytes were found to be not protected against oxidative stress after exposure to hydrogen peroxide, it is unlikely that these cells possess increased levels of antioxidants, such as glutathione, which are known to play a critical role in detoxification [49]. Instead, PAD4 has been reported to enhance NOX-dependent ROS production in human neutrophils by physically interacting with the cytosolic subunits NCF1 and NCF2. Knockdown of PAD4 abolished this interaction and reduced ROS generation by approximately 50% [50]. The contribution of NOX-dependent ROS generation to I/R injury is further supported by the findings of a previous study, showing strong protection against cardiac I/R injury in Nox1 and Nox2 knockout mice, which was widely preserved in isolated perfused hearts, suggesting an intrinsic mechanism independent on post-ischemic inflammation [51]. Given that our findings may indicate a similar intrinsic protective mechanism in cardiomyocytes, a potential regulation of NOX function by PAD4 appears entirely likely.

Moreover, NF-κB signalling has been implicated in promoting cell death in cardiomyocytes [52]. Strikingly, enhanced NF-κB activity increased mitochondrial ROS production in the left ventricle of obese mice [53] and both, genetic or pharmacological NF-κB inhibition, ameliorated reperfusion injury following MI through the inhibition of inflammation, ROS, and apoptotic and necrotic pathways [54–57]. PAD4 mediates the citrullination of the NF-κB subunit p65, promoting its nuclear translocation [58]. Accordingly, inhibition of NF-κB translocation in PAD4^-/-^ cardiomyocytes may partially contribute to reduced H/R- and I/R-induced cellular damage.

These previous findings in combination with our present data support the hypothesis that PAD4 inhibitors may represent a suitable strategy for preventing cardiac injury after I/R. Since MI is unpredictable and treatment at late reperfusion is no longer protective [59], only the administration of drugs just before reperfusion is of clinical relevance. Pre-treatment of cardiomyocytes with GSK484 before hypoxia mitigated H/R-related mitochondrial dysfunction and improved cell viability, although it did not reduce intracellular ROS formation. Moreover, administration of GSK484 only at the beginning of reperfusion neither prevented ROS formation nor ATP depletion at 4 h after reperfusion, but however it preserved cellular metabolic activity after 20 h of reoxygenation when compared to control cells. These results suggest that the protective effect of GSK484 administrated during the reperfusion phase is significantly weaker, likely due to limited exposure time. Indeed, a pre-incubation period of 20 min with the inhibitor has been proposed for effective PAD4 inhibition [12]. As we were unable to test whether GSK484 protects cardiomyocytes from H/R-induced injury when administrated shortly before reperfusion, this remains a limitation of our study. Additionally, the discrepancy between PAD4^-/-^ cardiomycytes and those treated with the inhibitor may point to the involvement of compensatory protective pathways, such as activation of the reperfusion injury salvage kinase (RISK) and survivor activator factor enhancement (SAFE) pathways [60,61]. Since these mechanisms were not explored in the present study, future investigations are needed to examine how PAD4 inhibition impacts cardioprotective signalling against I/R and H/R injury.

Taken together, our study uncovered a previously unrecognized, NETs-independent function of PAD4 in cardiomyocytes representing a novel, clinically relevant approach to reduce I/R-induced cardiac injury.

## Supporting information

S1 Table CF and LVDP of isolated pressure constant-perfused mouse hearts. Baseline: last min of stabilization period before ischemia/ reperfusion; CF: coronary flow; isch 5/25: 5/25 min of ischemia; LVDP: left ventricular developed pressure; n: number of mouse hearts; rep 10/30/60: 10/30/60 min reperfusion; Baseline values for CF and LVDP and their time courses were analyzed by two-way ANOVA for repeated measures and Fisher’s LSD post hoc test; *p < 0.05 vs. baseline, respectively.
(PDF)

S1 Fig Detection of NETs and neutrophils in WT hearts under baseline conditions. (A) For NETs detection, slices of WT hearts were stained with anti-MPO and anti-histone H3 (citrulline R2 + R8 + R17) antibodies, followed by the corresponding secondary antibodies. Nuclei were stained with DAPI. Representative immunofluorescence images from three mice are depicted. No NETs were detected under baseline conditions as demonstrated by the absence of MPO- and citrullinated histone H3 (citH3)-positive structures. Scale bar, 50 µm. (B) *Mpo* expression was quantified in WT hearts at baseline by real-time PCR (n = 6). (C) Quantification of neutrophils (CD45^+^CD11b^+^Ly6G^+^) in WT hearts under baseline condition by flow cytometry.
(PNG)

S2 Fig Visualization of PAD4 activation in H/R-exposed neonatal cardiomyocytes. Neonatal cardiomyocytes were exposed to hypoxia for 4 h followed by 0 h and 2 h of reoxygenation, respectively. In parallel experiments, cells were pre-incubated with 10 µM GSK484 2 h prior to hypoxia exposure to inhibit PAD4 activity. H/R-induced PAD4 activation was evidenced by the detection of citrullinated histone H3 (citH3) by immunofluorescence. Nuclei were counterstained with DAPI. The highest histone H3 citrullination was observed at 0 h following H/R. Representative immunofluorescence images from two independent experiments are depicted. Scale bar, 20 µm.
(PNG)

S1 Raw Images Original uncropped, unadjusted images from the western blot shown in Fig 3F. (PNG)
