# Supplementary material for: Peptidylarginine deiminase 4 deficiency alleviates hypoxia/reoxygenation-induced cardiomyocyte injury
Source: PLoS One. 2025 Sep 10;20(9):e0330864. doi: 10.1371/journal.pone.0330864 (PMC12422421; doi:10.1371/journal.pone.0330864)
Supplement: S1 Table — Baseline: last min of stabilization period before ischemia/ reperfusion; CF: coronary flow; isch 5/25: 5/25 min of ischemia; LVDP: left ventricular developed pressure; n: number of mouse hearts; rep 10/30/60: 10/30/60 min reperfusion; Baseline values for CF and LVDP and their time courses were analyzed by two-way ANOVA for repeated measures and Fisher’s LSD post hoc test; *p < 0.05 vs. baseline, respectively. (PDF) [file pone.0330864.s001.pdf]

S1 Table: CF and LVDP of isolated pressure constant perfused mouse hearts

	time	CF (ml/min)	LVDP (mmHg)
**WT** (n = 5)	baseline	3.2 ± 0.5	95 ± 10
	isch5	0.0 ± 0.0*	0 ± 0*
	isch25	0.0 ± 0.0*	1 ± 1*
	rep10	3.0 ± 1.2	20 ± 29*
	rep30	3.0 ± 1.2	39 ± 33*
	rep60	2.8 ± 1.0	42 ± 30*
**PAD4$^{-/-}$** (n = 8)	baseline	2.9 ± 0.6	98 ± 15
	isch5	0 ± 0*	3 ± 3*
	isch25	0 ± 0*	2 ± 3*
	rep10	2.8 ± 0.7	48 ± 39*
	rep30	2.7 ± 0.6	62 ± 33*
	rep60	2.6 ± 0.7	60 ± 26*
