# Supplementary figures and images for: Peptidylarginine deiminase 4 deficiency alleviates hypoxia/reoxygenation-induced cardiomyocyte injury
Source: PLoS One. 2025 Sep 10;20(9):e0330864. doi: 10.1371/journal.pone.0330864 (PMC12422421; doi:10.1371/journal.pone.0330864)

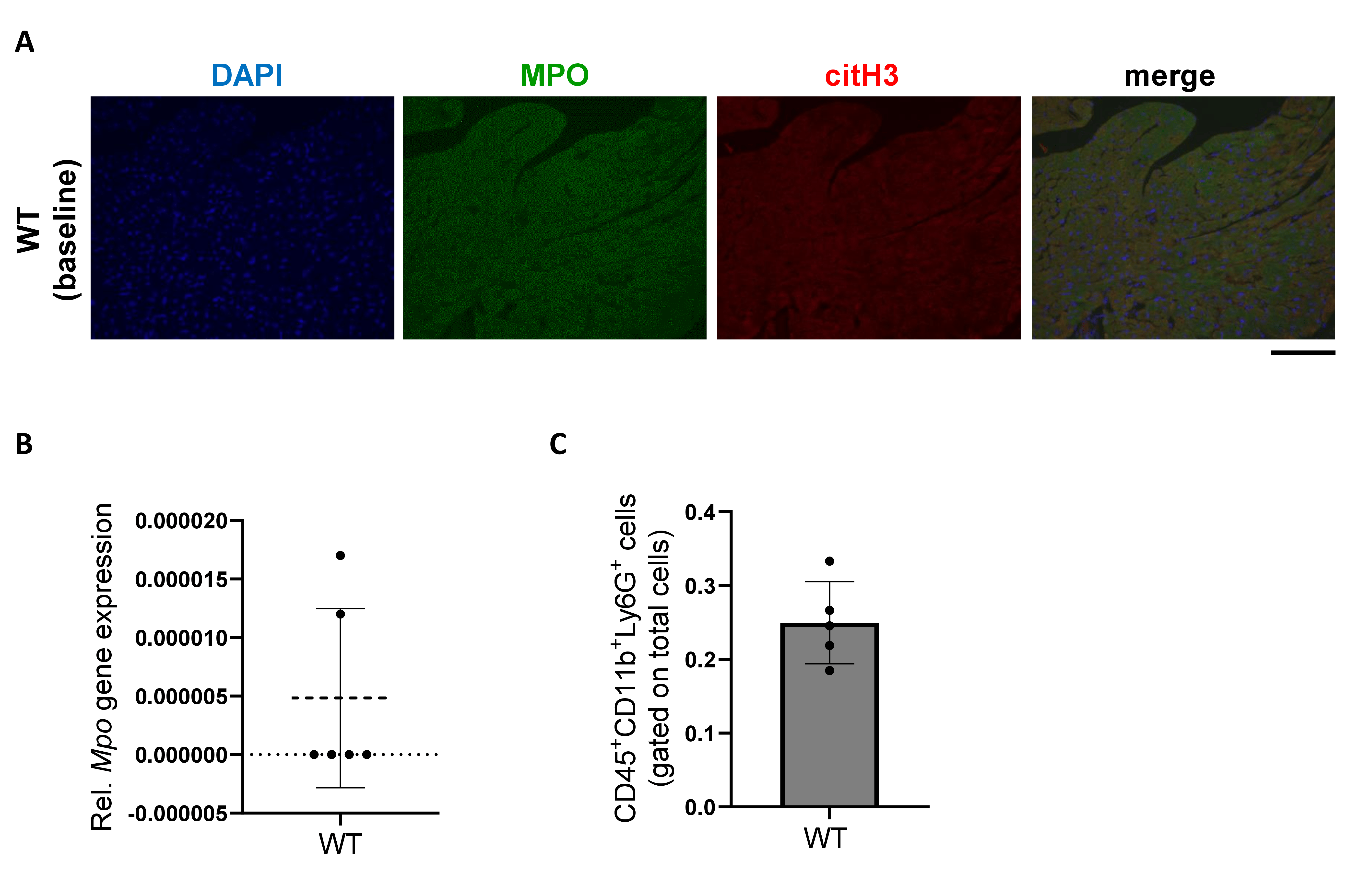

Supplement: S1 Fig — (A) For NETs detection, slices of WT hearts were stained with anti-MPO and anti-histone H3 (citrulline R2 + R8 + R17) antibodies, followed by the corresponding secondary antibodies. Nuclei were stained with DAPI. Representative immunofluorescence images from three mice are depicted. No NETs were detected under baseline conditions as demonstrated by the absence of MPO- and citrullinated histone H3 (citH3)-positive structures. Scale bar, 50 µm. (B) Mpo expression was quantified in WT hearts at baseline by real-time PCR (n = 6). (C) Quantification of neutrophils (CD45+CD11b+Ly6G+) in WT hearts under baseline condition by flow cytometry. (PNG) [file pone.0330864.s002.png]

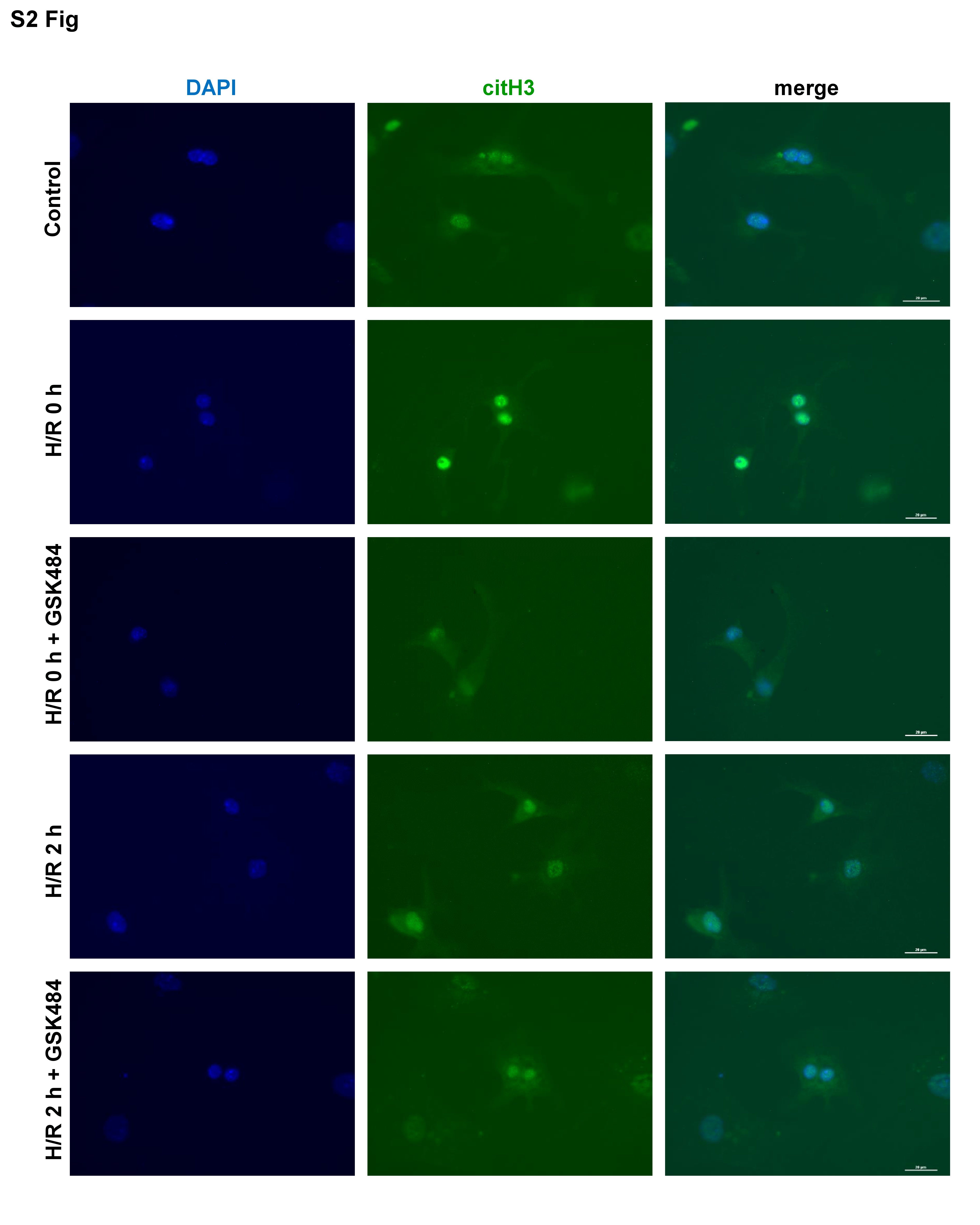

Supplement: S2 Fig — Neonatal cardiomyocytes were exposed to hypoxia for 4 h followed by 0 h and 2 h of reoxygenation, respectively. In parallel experiments, cells were pre-incubated with 10 µM GSK484 2 h prior to hypoxia exposure to inhibit PAD4 activity. H/R-induced PAD4 activation was evidenced by the detection of citrullinated histone H3 (citH3) by immunofluorescence. Nuclei were counterstained with DAPI. The highest histone H3 citrullination was observed at 0 h following H/R. Representative immunofluorescence images from two independent experiments are depicted. Scale bar, 20 µm. (PNG) [file pone.0330864.s003.png]

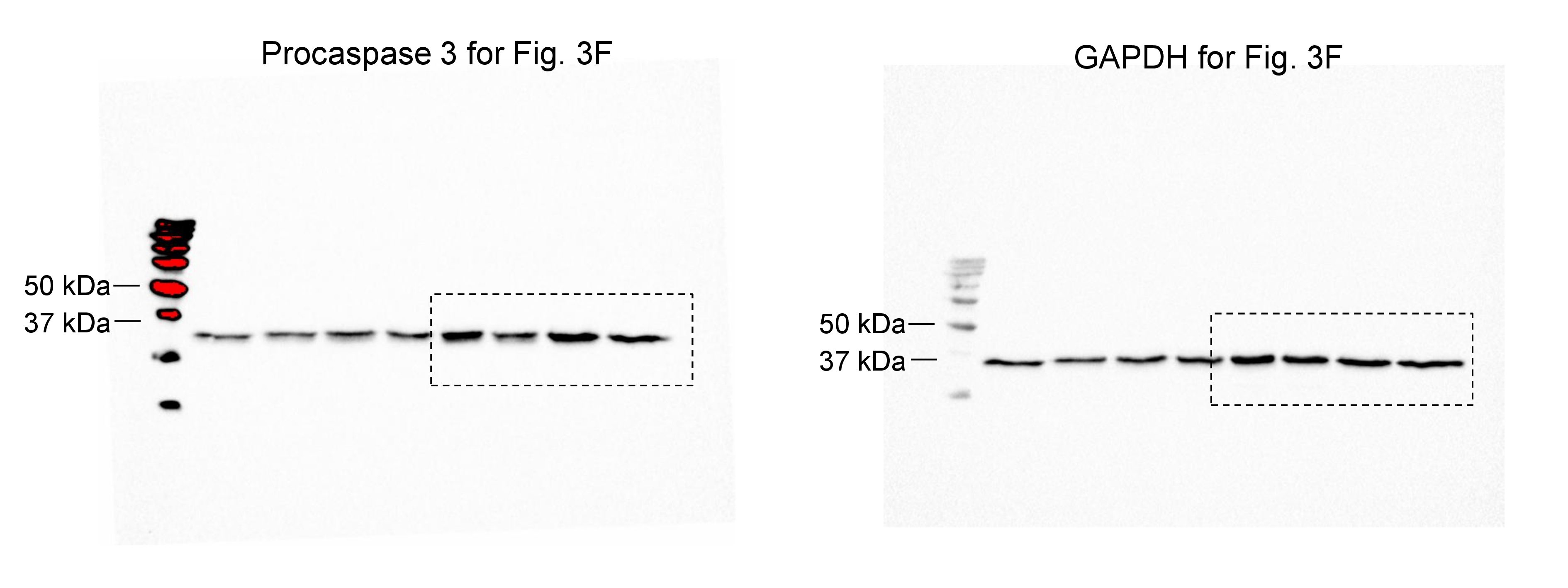

Supplement: S1 Raw Images — (PNG) [file pone.0330864.s004.png]
